# Supplementary material for: Locally Isolated Trichoderma harzianum Species Have Broad Spectrum Biocontrol Activities against the Wood Rot Fungal Species through Both Volatile Inhibition and Mycoparasitism
Source: J Fungi (Basel). 2023 Jun 15;9(6):675. doi: 10.3390/jof9060675 (PMC10301622; doi:10.3390/jof9060675)
Supplement: Supplementary file 1 [file jof-09-00675-s001.zip › Supplementary Figure S1.pdf]

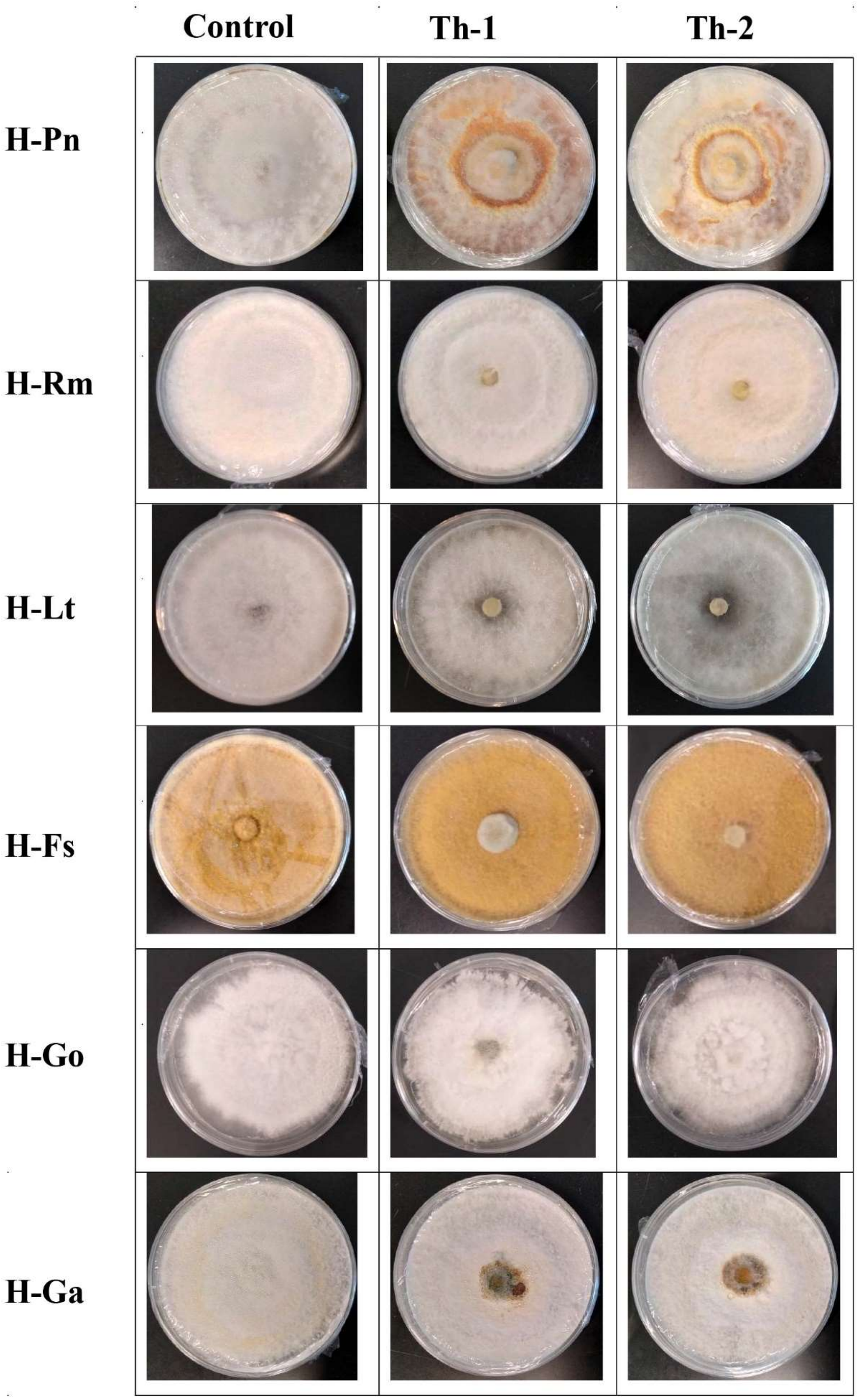

Supplementary Figure S1. Representative plates for cut plug assays 7 days after placing the *Trichoderma* plugs. Control plates had only pathogenic fungi growth without a *Trichoderma* plug in the centre. TH-1 and TH-2 are the two Trichoderma strains and H-PN, H-RM, H-LT, H-FS, H-GO and H-

GA were the six pathogenic fungi. Cut plug assay was modified from a protocol by Kamil et al. (Frontiers in Microbiology, May 2018, volume 9, article 829). Briefly, experiment plates were prepared by inoculating a pathogenic fungal plug to the center of a PDA plate. Once pathogenic fungal mycelia had fully covered the plate, a *Trichoderma* plug was inoculated to the center of the plate. The test was conducted in triplicates. Control plates lacked the inoculation of Trichoderma. All plates were incubated at 24°C and the ring of inhibition diameter was recorded on the seventh day.

For *Phellinus noxius* (H-PN), the mycelia outside the *Trichoderma* plug turned from white to brown and formed a distinct ring of inhibition. The effect of *Trichoderma* plug could be seen from Day 1, when browning in H-PN mycelia could be observed (Figure 6).

For *Rigidoporus microporus* (H-RM), the rings of inhibition were clearly visible 7 days after placing the Trichoderma plugs, equal sized for the two strains.

For *Fulvifomes siamensis* (H-FS), the dense white mycelia growth around the TH-1 plugs gradually spread over H-FS mycelia. There was no ring of inhibition for TH-2, however.

For H-LT (*L.asiodiplodia theoromae*), there was the lack of distinctive inhibition ring but thinner growth of its mycelia was observed, more so for TH-2.

H-GO (*Ganoderma orbiforme*) had a small ring of inhibition around the TH-1 plugs but appeared to have overwhelmed TH-2 with denser mycelia around the TH-2 plug.

H-GA (*Ganoderma australe*) formed a small ring of inhibition and produced a brown pigmented interaction zone surrounding the Trichoderma strain plugs, suggesting counter inhibition of the Trichoderma strains by the G. australe strain.
